# A case of hospital-acquired GNB infection - *P. aeruginosa* meningoencephalitis post laparoscopic cholecystectomy for biliary pancreatitis, complicated with portal vein branch thrombosis and intracerebral ischemic and hemorrhagic lesions

**DOI:** 10.1186/1471-2334-14-S7-P98

**Published:** 2014-10-15

**Authors:** Andrei Rogoz, Raluca Zlotea, Cleo Roşculeț, Cătălin Apostolescu, Marius Radu, Liana Gavriliu, Georgeta Ducu, Otilia Benea, Doina Iovănescu

**Affiliations:** 1 National Institute for Infectious Diseases "Prof. Dr. Matei Balş", Bucharest, Romania

## Background

*Pseudomonas aeruginosa* infections involving the CNS usually present as meningitis or brain abscesses. The CNS invasion is the result of direct inoculation (head trauma, surgery), spread from a distant site (urinary, abdominal infections) or by direct invasion of a contiguous structure (inner ear, head sinus).

## Case report

We present the case of a female patient admitted to our clinic with a suspicion of acute bacterial meningoencephalitis, one month after a laparoscopic cholecystectomy. During the first 48 hours she presented generalized seizures, 5-6 daily, with a duration that ranged from 30 to 60 seconds, that responded to medical therapy. The CSF cultures and the pulmonary tract secretions both tested positive for *P. aeruginosa*. The antibiotic regimen consisted of iv meropenem, colistin and ciprofloxacin for 7 days, then meropenem and ciprofloxacin for 21 days. The evolution and the treatment decisions were complicated by the discovery on the cerebral MRI of bilateral frontal ischemic and hemorrhagic lesions and a portal vein branch thrombosis.

The patient registered almost complete cognitive and motor recovery, and is continuing the kinetotherapy.

## Conclusion

This *P. aeruginosa* isolate had a resistance profile that permitted the use of antibiotics with good CNS penetration, which proved a decisive factor in the therapeutic success.

## Consent

Written informed consent was obtained from the patient for publication of this Case report and any accompanying images. A copy of the written consent is available for review by the Editor of this journal.

